# Genetic Animal Models of Idiopathic Generalized Epilepsies: What Can We Learn from Them?

**DOI:** 10.3390/biomedicines13061301

**Published:** 2025-05-26

**Authors:** Bernard Lakaye, Laurent Nguyen

**Affiliations:** 1 Laboratory of Molecular Regulation of Neurogenesis, GIGA Institute, University of Liège, CHU Sart Tilman, 4000 Liège, Belgium; lnguyen@uliege.be; 2 WELBIO Department, WEL Research Institute, Avenue Pasteur 6, 1300 Wavre, Belgium

**Keywords:** idiopathic generalized epilepsy, spike and wave discharges, generalized tonic–clonic seizures, thalamocortical network

## Abstract

The use of animal models of idiopathic generalized epilepsy (IGE) is of great importance in the field of epilepsy research, with IGE affecting more than 20 million people worldwide. IGEs are characterized by a high degree of genetic heterogeneity, which makes it difficult to understand the underlying mechanisms leading to seizures. The development of animal models, whether spontaneous or resulting from genetic manipulation, has significantly contributed to our understanding of the pathological processes underlying certain IGEs, notably absence epilepsy. Research suggests that the concept of generalized epilepsy covering the whole brain should be replaced by a model in which the thalamus and its various nuclei are integrated into thalamo-cortical loops. These then assume distinct roles in the generation and generalization of seizures, which may differ across the spectrum of IGE disorders. The study of epileptogenesis is also essential: this area of research, grounded in systematic developmental neuroscience, examines the intermediate stages of neuronal activity to determine when, and how, functional development diverges between healthy and pathological states. Understanding nervous system development requires a comprehensive view of how anatomic, molecular, and genetics factors relate to neuronal activity. The emerging use of optogenetic methods and human assembloids will greatly aid our understanding of the mechanisms underlying these processes.

## 1. Introduction

Seizures are among the most common neurological disorders, affecting people of all ages, from newborn to the elderly. They are to some extent unique in their diversity and complexity. Indeed, following the ILAE classification, 41 epileptic syndromes have been characterized based on seizure type, epilepsy type, etiology, and co-morbidities [1]. Among these, Idiopathic Generalized Epilepsies (IGEs) form a distinct “historical” subgroup of Genetic Generalized Epilepsy (GGE), a group of epilepsies characterized by generalized seizures, which also includes developmental and epileptic encephalopathies (DEEs) [2]. IGEs and DEEs differ in several aspects: First, in terms of etiology, since the causal mutations for many DEEs are known. Secondly, in terms of age of onset: most DEEs begin during the neonatal period to young adulthood. Thirdly, in their response to medication, as DEEs are often refractory to antiepileptic drugs (AEDs). Fourthly, in terms of comorbidities, because DEEs are often accompanied by developmental delay and encephalopathy. Moreover, IGEs are the most common syndromes within the GGEs, accounting for approximatively 15 to 20% of people suffering from epilepsy [2]. The generic IGE term encompasses the following syndromes: Childhood Absence Epilepsy (CAE), Juvenile Absence Epilepsy (JAE), Juvenile Myoclonic Epilepsy (JME), and Generalized Tonic–Clonic Seizures Alone (GTCSA).

The genetic basis of IGE was first revealed in a study by Lennox, who compared the degree of concordance of these epileptic syndromes in pairs of monozygotic and dizygotic twins and showed that it was higher in monozygotic twins than in dizygotic twins [3]. Despite this early evidence and the significant progress made in genetic analysis, identifying the genes responsible for these IGEs remains challenging. This is because the inheritance pattern does not follow a simple Mendelian pattern of autosomal dominant/recessive alleles. Inheritance in IGEs is complex due to the polygenic nature of these diseases and the possible epigenetic influences [4].

Despite these challenges, important insights into the mechanisms underlying IGEs have been made possible through the study of animal models that closely resemble the human counterpart. Brief information about the IGE models described in this review is given in Table 1.

## 2. Childhood Absence Epilepsy (CAE)

### 2.1. Characteristics of CAE in Humans

CAE affects around 2–8% of epileptic patients with symptom onset typically between 5 and 8 years of age. For two thirds of patients, absence seizures (ASs) resolve by mid-adolescence while for others, additional epilepsy syndromes, such as JME, may develop as well. Absence seizures (ASs) are characterized by sudden and brief lapses of consciousness lasting about 10–20 s, without voluntary movements. ASs occur on average 10–50 times a day but the frequency can sometimes reach up to 200 times per day. On EEG analysis, typical ASs are associated with bilateral, synchronous, and regular 3 Hz spike-and-wave discharges (SWDs). The first line antiepileptic drug (AED) for treating CAE is ethosuximide (ETX) but valproate (VPA) or lamotrigine or a combination of the two may also be recommended. If these treatments are ineffective, topiramate or leviteracetam may also be prescribed.

Brain imaging studies have revealed a bilateral reduction in gray matter (GM) volume in the thalamus and in the temporal lobes of CAE patients [5,6,7]. A cross-sectional study reported that age-related changes in cortical thickness and sulcal depth of several lobes differed between CAE patients and controls, possibly reflecting either a developmental delay or irreversible abnormalities in the pruning process [8]. White matter (WM) analysis using diffusion tensor imaging (DTI) shows reduced fractional anisotropy (FA) in the genu of the corpus callosum [9]. Although CAE was historically considered as a benign form of epilepsy, recent clinical studies indicate that ASs are associated with cognitive comorbidities such as memory deficits, executive dysfunction, and impairments in visual perceptual skills [10].

Although the genetic basis of CAE is well established, identifying specific disease-associated genes has proved difficult, largely because CAE is a polygenic disorder with incomplete penetrance and likely compounded by the inclusion of patient cohorts with complex phenotypes in many genetic studies. Early genetic studies relied on the direct sequencing of candidate genes linked to SWDs. Although this approach identified mutations in several subunits of the GABA_A_ receptor, they have only been detected in a limited number of patients [11]. Among the three genes encoding T-type calcium channels, no mutations have been reported for Cav3.3, one has been found in Cav3.1, and around ten missense mutations have been identified in Cav3.2, some of which resulted in a gain-of-function effect [12,13,14]. Finally, although hyperpolarization-activated cyclic nucleotide (HCN) channels play a key role in SWDs, only three CAE patients have been reported to carry mutations in the HCN1 channel [15]. In 2018, the first GWAS study of a purely CAE cohort uncovered two significant loci (2p16.1 and 2q22.3) and prioritized, within these regions, three genes (*FANCL*, *BCL11A*, and *ZEB2*) that are not linked to membrane channels or transmitter pathways [16]. In their latest study, the ILAE consortium confirmed *BCL11A* as a strong candidate gene for CAE [17].

### 2.2. The Thalamo-Cortical Loop: The Key Player in Absence Seizures

The feline penicillin model was the first to provide evidence that 3–4 Hz SWDs require the functional and anatomical integrity of both the thalamus and the cortex. Subsequent research on rodent models has refined the circuitry involved and identified the molecular players responsible for SWDs generation [18]. The thalamocortical loop (TC-loop), which generates SWDs, consists of three main neuronal players, the thalamocortical (TC) neurons, the corticothalamic (CT) neurons, and the nucleus reticularis (nRT) neurons. TC neurons of the ventrobasal thalamus (VB) are glutamatergic projections neurons that innervate layer 4 cortical neurons and receive glutamatergic input from CT neurons in cortical layer 5/6. The nRT neurons are GABAergic and form a distinct structure situated between the two projection nuclei, called the nucleus reticularis thalami. Both TC and CT neurons also send glutamatergic axon collaterals to the nRT. TC and nRT neurons can fire in two different modes: tonic during wakefulness and burst during sleep or seizures. Tonic firing occurs when the membrane of TC neurons is at resting potential (−55 mV) and burst firing occurs when the membrane is hyperpolarized (−70 mV). Once TC neurons are hyperpolarized, which can occur if multiple nRT neurons fire together to produce strong IPSPs, their T-type Ca^2+^ channels and HCN channels are activated, producing a burst of high-frequency action potentials (APs). These APs reactivate the nRT and activate CT projection neurons. In turn, CT neuron firing can combine with TC output to activate nRT cells. This three-cell-type model provides a framework for understanding the coordination of the thalamocortical network during SWDs [19].

### 2.3. Spontaneous Models of Absence Epilepsy in Mammals

#### 2.3.1. Monogenic Mutant Mouse Models

At least six mutant strains arose spontaneously from inbred mouse strains (some at the Jackson Laboratory) and breeding studies confirmed that the phenotype of each of these mutants was due to a single recessive mutation. Notably, all these mutant mice exhibited additional behavioral and developmental abnormalities in addition to ASs, a feature rarely, if ever, observed in human CAE. Below, we will limit our description to the four most extensively studied models, as follows:

- Tottering mouse

The tottering (*tg*) locus on chromosome 8 encodes for the α1A subunit of the voltage-gated Ca^2+^ channel (CACNA1A), the core subunit of Cav2.1 (P/Q-type) calcium channels. A point mutation (P601L) in the gene has been shown to underlie the phenotype in this mouse model, reducing the Cav2.1 current density in Purkinje neurons by approximatively 40% [20]. The phenotype of this mouse line includes ataxia, paroxysmal dystonia (motor dysfunction with normal EEG), and absence seizures with 5–7 Hz SWDs. Seizure onset begins at three weeks of age while ataxia becomes evident after four weeks. At the cellular level, *tg* mutants exhibit several defects, including a loss of both cerebellar granule and Purkinje cells. Additionally, a reduction in forebrain and hindbrain weight, as well as a reduction in the thickness of the molecular layer in the paramedial lobule of the cerebellum are observed; however, this is apparent only after the onset of behavioral symptoms [21]. Another notable cytological alteration is the hyperinnervation of the CNS by noradrenergic fibers from the locus coeruleus [22].

- Stargazer mouse

The stargazer (*stg*) locus on chromosome 15 encodes for the γ2 subunit of the voltage-gated Ca^2+^ channel (CACNG2), also known as “stargazin”. A transposon insertion into intron 2 of the gene leads to premature transcription termination [23]. Although the γ2 subunit has a minor effect on VDCC activity, it plays a crucial role in AMPA receptor (AMPAR) trafficking and synaptic targeting. In stargazer mice, reduced AMPA synaptic currents are recorded in many brain structures including the cerebellum and thalamic reticular nucleus [24,25]. A reduced expression of the GluA4 subunit of AMPAR in paravalbumin-positive (PV+) interneurons of the somatosensory cortex is apparent before seizure onset, whereas GluA2 expression declines only after seizure onset. This suggests a differential contribution of different GluA subunits within AMPAR to seizure generation and maintenance [26]. The mutant mice are recognizable at P14 by their smaller body size and ataxic gait. Symptoms will worsen with age, although mutants typically survive beyond one year of age, but they exhibit severe motor impairments including poor rotarod performance and the inability to swim [27].

- Lethargic mouse

The lethargic (*lh*) locus on chromosomes 2 encodes for the ß4 subunit of the voltage-gated Ca^2+^ channel (CACNB4). A four-nucleotide insertion into a splice donor site results in a truncated protein [28]. The phenotype consists of the emergence of severe ataxia and lethargy at 15 days of age, followed closely by focal motor seizures and a second seizure type consisting of brief behavioral immobility accompanied by generalized SWD. The lh mutants are smaller and weaker than controls and suffer from thymic involution at 3–4 weeks of age with a concomitant reduction in cell-mediated immunity. Most mutants die before 2 months of age, with loss of the ß4 subunit inducing a compensatory reshuffling of ß-subunits, which is associated with a reduced expression of the N-type Ca^2+^ channel in both the forebrain and cerebellum [29]. Increased GABA_B_ receptor binding densities have also been reported in the neocortex of lethargic mice [30].

- Slow-wave epilepsy mouse

The slow-wave epilepsy (*swe*) locus on chromosome 4 encodes for the ubiquitous sodium hydrogen exchanger (NHE1). In the swe mice, a point mutation introduces a premature stop codon after amino acid 441, located between transmembrane segment 11 and 12 [31]. This mutation causes mice to develop ataxia between P11 and P14, and is associated with a high mortality rate; most *swe* animals die after 35–40 days. In the rare event that mutants survive beyond 40 days, EEG recordings reveal burst activity of 3 Hz SWDs associated with behavioral arrest beginning around 4–5 weeks. Interestingly, these seizures resolve with time, as they were not detected in 6-month-old mice, and are influenced by the genetic background, making it the only rodent model whose absence seizures disappear with age [31].

#### 2.3.2. Polygenic Rat Models

- GAERS model

The “Genetic Absence Epilepsy Rats from Strasbourg” (GAERS) originates from the breeding of Wistar rats presenting spontaneous absence seizures [32]. In this strain, 100% of the animals experience recurrent generalized non-convulsive seizures. These bilateral synchronous SWDs are accompanied by behavioral arrest, staring, and, often, twitching of the vibrissae and facial muscles. Interestingly, during SWDs, GAERS consistently pause lever pressing for obtaining food, suggesting a temporary disconnection from their environment [33]. Interestingly, SWDs are first detected around 30–40 days of age, although abnormal oscillations in the somatosensory cortex are observed as early as 14 days postnatally [34]. The number of SWDs appears to reach a plateau at approximatively 4 months of age, persisting until the death of the animal, with a frequency of approximately 7–11 Hz [33]. Ex vivo diffusion tensor imaging (DTI) revealed a decreased callosal fractional anisotropy (FA) while volumetric MRI detected increased volumes of the amygdala, cortices, and ventricles, along with a thickening of the somatosensory cortex [35,36]. Interestingly, BOLD-fMRI signals in awake GAERS resemble those seen in humans, showing decreased activity in the primary somatosensory cortex (S1) and an increased activity in the VB thalamus during absence seizures [37].

The GAERS and their non-epileptic control (NEC) strain are fully inbred. Through breeding experiments, it was first established that the inheritance pattern of SWDs is dominant, and that this inheritance is polygenic with three quantitative trait loci on chromosomes 4, 7, and 8 influencing the frequency, amplitude, and duration of SWDs [38]. Two mutations were first identified in the GAERS. The first is an additional alanine residue in the polyalanine tract of the C-terminal intracellular domain of KCNK9 (chr 15). However, this mutation has no effect on channel expression and has no apparent functional consequences in vivo or in vitro [39]. The second one is a point mutation (R1584P) in the *Cacna1h* gene (Chr 10), which encodes Cav3.2, one of the three T-type calcium channels [40]. While the total Cav3.2 mRNA copy number remains unchanged, GAERS exhibit an altered ratio of a splice variant in the thalamus. The splice variant mostly expressed in GAERS underlies a gain-of-function effect, with the channel recovering faster from inactivation while it also generates increased charge transference during high frequency bursts [40]. However, introducing the R1584P mutation into the NEC background (i.e., the seizure-resistant background) does not produce absence seizures [41]. In fact, whole genome sequencing of GAERS and NEC strains has identified a substantial number of single nucleotide variants (SNVs), short insertion-deletions, and potential copy number variations that result in complete or partial loss/duplication of 41 genes. Among these variants, 25 variants lead to stop codon gain/loss, 56 affect putative essential splice sites and 56 are indels predicted to result in frameshifts. These genetic variations likely contribute to the complex inheritance of the seizure phenotype and the resistance, or susceptibility, of both strains to seizures [42]. By performing specific crosses and comparing sequencing data with rat-specific databases, 6 GAERS-specific SNVs and 14 NEC-specific SNVs have been identified [42].

At the cellular and molecular levels, many alterations have been identified in the thalamo-cortical circuit responsible for the generation of the SWDs. Notably, even before seizure onset, low-threshold T-type calcium currents (but not L-type) amplitudes are increased, and GABA_A_ IPSCs exhibit larger amplitude and faster decay in the nRt of the GAERS. These changes are absent in both the cortex and VB [43,44]. Additionally, while hyperpolarization-activated current (I_h_) is not altered in TC neurons, its sensitivity to cAMP is reduced before seizure appearance, and this persists during the chronic state [45]. This may be linked to the upregulation of HCN1 mRNA, the cAMP-insensitive channel, in both the ventroposterior thalamus and nRT, while the transcription of HCN2 and 4, both cAMP-sensitive, remains unchanged [45]. Further molecular analysis has shown an increased expression of Stargazin, GluA1, and GluA2 in the cortical membrane fraction of epileptic but not juvenile GAERS [46]. Examination of the GABA system has revealed a decreased expression of ß2-ß3 GABA_A_ subunits in the sensorimotor cortex and anterior thalamic area [47]. However, studies on GABA_A_ receptor binding sites have only identified differences in the CA2 region of the hippocampus [48]. No neuronal loss has been observed in any of the three structures contributing to SWDs [49]. However, while initial reports suggested that the density or distribution of interneurons were not disturbed, a more recent and specific study revealed a significant increase in PV+ interneuron density in the S1 somatosensory cortex and decreased density in the hippocampal hilus [47,50].

Although SWDs are generated by neuronal activity, changes involving astrocytes have also been documented, often preceding the onset of seizures. The first reported alteration was an increase in GFAP expression in the cortex and thalamus prior to seizure onset [51]. Subsequent studies confirmed this finding and extended it to all thalamic subnuclei examined, i.e., the nRT, the VB, the centromedial, and the dorsal lateral geniculate [52]. Interleukin-1ß (IL-1ß) is expressed in activated astrocytes of the somatosensory cortex when immature types of SWDs begin to emerge [53]. Furthermore, in the cortex, the expression of the astrocytic glutamate transporters (i.e., GLT-1 and GLAST) is decreased before seizures but returns to normal in adult GAERS [54]. Surprisingly, primary cultures of cortical astrocytes from newborn GAERS exhibit reduced glutamate uptake and GLAST protein expression, suggesting a cell-autonomous defect [54]. Finally, impaired astrocytic modulation of thalamic GABA concentration, associated with a malfunction of the astrocytic GAT-1, is observed [55].

Influences and changes in the brain regions beyond the TC-loop have been reported in GAERS, as the thalamus is extensively connected to other brain regions. In the basal ganglia, recordings of substantia nigra pars reticulata (SNpr) show a strong correlation between SWDs and the burst activity of APs, which terminate at or before SWDs cessation [56]. Moreover, the administration of dopamine D1/D2 agonist or antagonist into the nucleus accumbens core reduces or enhances absence seizures, respectively, without behavioral or EEG side effects [57]. Investigations into the cerebellum were limited in the GAERS before the development of optogenetic techniques, but one study reported a decreased expression of Cav2.3 mRNA exclusively in the epileptic GAERS, though the relevance for ASs was not investigated further [58].

- WAG/Rij model

The Wistar–Albino–Glaxo from Rijswijk (WAG/Rij) is a rat strain of Wistar origin that has been extensively used and validated as a genetic model of generalized absence epilepsy. In this epileptic rat strain, 100% of the animals present generalized non-convulsive seizures with behavioral episodes during SWDs, similar to those observed in GAERS [59]. However, there are significant differences between both strains. First, WAG/Rij rats exhibit two types of SWDs: type 1 SWDs (7.5–9.5 Hz, lasting 3–4 s) are generalized, bilaterally symmetrical, and observed in all animals. In contrast, type 2 SWDs (8 Hz, lasting ±1 s) are localized in the occipital–parietal area and occur in about 60% of the animals [60]. Moreover, the number, mean duration, cumulative duration, and frequency of SWDs is lower in the WAG/Rij, while discharge frequency is higher [61]. Additionally, seizure onset occurs later in WAG/Rij, after P50, and both the duration and frequency of SWDs increase with age [62].

Diffusion tensor imaging has revealed significantly decreased FA and increased perpendicular diffusivity in the anterior part of the corpus callosum, but only in epileptic WAG/Rij [35]. Functional MRI shows a strong connectivity between the brain regions involved in ASs suggesting that, over time, ASs induce lasting changes in the epileptic network even during the resting state [63].

In WAG/Rij, two quantitative trait loci (QTL) have been identified on chromosomes 5 and 9 as regions controlling type 2 and type 1 SWDs characteristics, respectively [64]. However, so far, no genomic mutations have been identified in this model.

Given the importance of current I_h_ pacemakers in the maintenance of neuronal rhythmicity and the generation of SWDs, several studies have investigated the subunits of the channels responsible for generating this current. In the somatosensory cortex of epileptic rats, the fast component of I_h_ is reduced by 70% with a corresponding decrease in HCN1 channel subunit expression over time, which begins prior to the onset of spontaneous seizures [65,66]. Loss of HCN1 in the apical dendrites of layer 5 pyramidal neurons of the somatosensory cortex increases the somato–dendritic coupling, thereby lowering the frequency threshold required to generate dendritic Ca^2+^ spikes via backpropagating APs [66]. Conversely, in TC neurons, HCN1 expression is elevated, resulting in an increased I_h_ current density, though its properties are partly changed. Specifically, a negative shift in the activation curve and an altered cAMP responsiveness have been observed; this has been attributed to the expression of a shorter form of HCN1 (containing an N-terminal deletion of 37AA) which is encoded by mRNA but not by genomic DNA [67]. When expressed in *Xenopus* oocytes, this variant leads to a 2-fold increase in the I_h_ current amplitude while simultaneously reducing the HCN2 and HCN4 currents in co-expression experiments [67]. Additionally, studies have demonstrated an increased T-type current density in the epileptic WAG/Rij rats, consistent with an elevated mRNA expression of the corresponding channel in three thalamic nuclei [68]. In both pre-epileptic and epileptic WAG/Rij rats, the expression of the NR1 subunit of the NMDA receptor and the GluA4 subunit of the AMPA receptor is reduced in layers 4, 5, and 6 of the peri-oral somatosensory cortex [69]. The expression of the metabotropic glutamate receptor mGluR4, which has been proposed to be neuroprotective, is lower in the nRT of pre-epileptic rats but higher in epileptic individuals of the same strain [70,71]. Several changes in the expression of GABA receptors have also been reported in this strain, notably, an increased expression of the α4 and δ subunits of the GABA_A_ receptor have been reported in the nRT of epileptic animals [72]. Additionally, changes in GABA_B_ receptor subunit expression and distribution have been documented in the cortex and distal dendrites of neocortical pyramidal cells [73].

Historically, absence epilepsy was considered to be a purely functional disorder, so morphological studies were relatively rare until recently. In the parietal and forelimb area of the somatosensory cortex of six months old WAG/Rij rats, the density of PV+ interneurons is approximatively half that observed in the non-epileptic control strain [59]. Furthermore, in the upper layers of both the somatosensory and motor cortex, pyramidal cell distribution is disorganized, and dendritic properties differ from those of non-epileptic controls (e.g., apical dendrites are not perpendicular and often split into two branches) [74]. Increased GFAP expression has been detected in the somatosensory cortex, nRT, and VB of the epileptic animal [52]. Changes in the glia to neuron ratio have been observed in specific layers of the somatosensory, motor, and cingulate cortex of the epileptic WAG/Rij; however, only in the motor cortex is this change attributed to a reduced number of neurons [75]. In this model, a recent study reported that the oligodendrocyte number and axonal myelination within the seizure circuit increased after seizure onset. Notably, the blockage of seizures by ETX treatment from 1.5 months of age, i.e., before seizure onset, until 7 months of age normalized myelin sheath thickness [76]. Based on these findings, and supported by additional experiments in which activity-dependent oligodendrogenesis and myelination were conditionally suppressed, it was suggested that the abnormal increase in myelination within the thalamocortical network (responsible for maintaining its excessive synchrony), is the result of activity-dependent myelination. These findings validate the well-known adage in epilepsy research: “seizures beget seizures” [76].

### 2.4. Models of Absence Epilepsy Based on Genetic Manipulation of Candidate Genes

With the development of methods to manipulate the genome, numerous knockout mouse models of previously identified candidate genes have been generated to test the current hypothesis in the mechanisms controlling the TC-loop and generation of SWDs.

The role of GABA neurotransmission for the control and generation of SWDs is well established; however, results from knockout mice affecting different receptor subunits or types are complex. In *Gabra3*-KO mice, no spontaneous SWDs are recorded, but pharmacologically induced absence seizures display reduced duration and power. In contrast, *Gabrb3*-KO mice occasionally exhibit absence seizures [77,78]. *Gabbr1* and *Gabbr2*-KO mice show rare or no spontaneous SWDs, respectively [79,80]. As predicted from enhanced tonic inhibition in the thalamocortical neurons of GAERS, due to compromised GAT-1 activity, GAT-1 knockout mice do present spontaneous, ETX-sensitive SWDs [81].

T-type calcium currents which are critical for SWDs generation are mediated by three different channels, each with distinct brain expression profiles. Knockout mice lacking the *Cacna1g* gene (encoding Cav3.1) lack the burst firing mode in the nRT and are resistant to baclofen and GHB-induced seizures [82]. Conversely, whole brain overexpression of Cav3.1 increases T-type currents in thalamic neurons and induces ETX-sensitive ASs [83]. In the *tg*, *lh*, and *stg* mice’s nRT, T-type currents are greater than in control mice [84] but, in an Cav3.1-KO background, their spontaneous SWDs are either completely suppressed (for *tg*) or strongly reduced (for *lh* and *stg*) [85]. In *Cacna1i* (Cav3.3) knockout mice, despite a loss of over 70% of T-type currents in the nRT (residual current being driven by Cav3.2), susceptibility to GHB-induced seizures is increased [86]. Unexpectedly, GHB remains as effective on the Cav3.2 and Cav3.3 double-KO mice despite a complete absence of bursting activity in the nRT [86].

Several mouse lines targeting different isoforms of HCN channels have been developed. Surprisingly, constitutive *Hcn1*-KO mice do not exhibit spontaneous ASs and, although their susceptibility to drug-induced ASs was not tested, they do show an increased sensitivity to acute and chronic convulsive seizures [87,88]. In contrast, *Hcn1*-KO rats display spontaneous ETX-sensitive ASs that increase in frequency and duration with age [89]. This discrepancy may be due to differences in genetic background, as F344 rats are predisposed to spontaneous Ass, whereas certain knockout mouse models (such as *Gria4*-KO mice, for example) exhibit a variable ASs phenotype dependent on genetic context [90]. Conversely, *Hcn2*-KO mice express spontaneous ETX-sensitive ASs, with a significant reduction in I_h_ currents in the TC neurons of the VB, and a complete absence of I_h_ currents in the nRT [91,92]. No spontaneous ASs have been recorded in *Hcn4*-KO mice [93].

Recent studies have shown that tamoxifen-induced ablation of *Cacna1a* in the adult stages recapitulate the neurological phenotype of the inborn-deficient mice [94]. This deletion does not promote a T-type current in nRT; however, T-type currents remain important, as mice generated in a *Cacna1g*-KO background exhibit reduced seizure susceptibility [94]. The specific ablation of *Cacna1a* in layer 6 pyramidal neurons–the only layer projecting to the nRT and VB–results in robust spontaneous ETX-sensitive SWDs, accompanied by an increased T-type Ca^2+^ current in both thalamic nuclei [95]. Additionally, the knockdown of *cacna1aa*, the cacna1a paralog highly expressed in zebrafish brains, induces hypoactivity and epileptiform-like EEG events, which are reduced by ETX, VPA, lamotrigine, and topiramate [96]. The *Nhe1*-KO mice reproduce the phenotype of the *swe* mice, with a large proportion of animals experiencing early mortality (between P16 and P29), ataxia, and epileptic-like seizures, also EEG recordings have not yet been performed [97]. In these mice, the Na+ current density is increased in both the CA1 and cortex due to the upregulation of the Na+ channel subtype I in the CA1 area and subtype II in the cortex [98]. Unfortunately, these mice have not been analyzed beyond six months of age.

### 2.5. Optogenetic and Chemogenetic Models to Manipulate ASs

Optogenetic and Designer Receptors Exclusively Activated by Designer Drugs (DREADDs) approaches allow real-time manipulation of specific neuronal or glial populations, providing unprecedented insights into the network complexities, and their potential critical choke points.

In the WAG/Rij rats, the activity of the deep and intermediate layers of the superior colliculus decreases in the seconds prior to SWDs onset but increases during the SWDs. Excitatory optogenetic simulation effectively reduces seizures in only males when applied in an open-loop (continuous) neuromodulation approach, whereas a closed-loop (on-demand) configuration is effective in both sexes [99]. In the gamma butyrolactone (GBL) model of absence epilepsy, the bilateral stimulation of the dorsal striatum attenuates SWDs manifestations in both open- and closed-loop configurations, whereas bilateral inhibition increases the number of SWDs [100]. These findings confirm the role of the extended basal ganglia in ASs control, although they do not clarify whether direct, indirect, or both pathways are involved.

The cerebellum has an extensive network of outputs projecting directly to various thalamic nuclei and indirectly to cortical regions. The first experiment demonstrating that optogenetic activation of cerebellar neurons modulate SWDs was conducted in a *tg* mouse model [101]. Subsequent studies revealed that specific inactivation of *Cacna1a* in the cerebellar granule cells (*quirky* mouse) or Purkinje cells (*purky* mouse) leads to recurrent SWDs. In both models, increasing cerebellar nuclei (CN) activity via the activation of G_q_-coupled DREADDs reduces SWDs incidence, whereas decreasing CN activity via G_i/o_-coupled DREADDs increases SWDs incidence [102] Furthermore, a one-second-long, closed-loop optogenetic stimulation of CN is sufficient to abort SWDs in both models [102]. In a recent model of absence epilepsy and dyskinesia, the BK-D434G mice (which harbor a gain-of-function mutation in the *Kcnma1* gene, encoding the pore-forming α subunit of the Ca^2+^- and voltage-activated large conductance BK-type potassium channels), a new nucleus was identified as a modulator of ASs [103]. In these mice, *c-fos* expression is increased in the midline thalamic nucleus (MLT) but not in the nRT or in the ventral posteromedial nucleus, a component of the VB. The optogenetic activation of MLT neurons prevents their bursting, thereby suppressing SWDs generation and rescuing the animals from seizure-induced arrest and vigilance deficits [103].

As discussed earlier, in several ASs models, accumulating evidence suggests that astrocytes play a role in neuronal activity regulation. In spontaneous absence epilepsy models, changes in astrocytes are observed even before the appearance of an epileptic phenotype. The optogenetic manipulation of astrocytes in the VB thalamic nuclei influences SWDs in a model-dependent manner. In GAERS, stimulation increases the SWDs duration without affecting their number, whereas in WAG/Rij, it increases SWDs number without altering their duration, confirming in both cases the importance of astrocytes in modulating the SWDs [104].

Local chemogenetic inhibition of PV+ interneurons in cortical (somatosensory cortex) or thalamic feedforward (nRT) microcircuits induces SWDs oscillations and behavioral arrests [105]. Conversely, DREADDs-mediated local activation of PV+ neurons in the same regions prevented PTZ-induced absence seizures [106]. These findings highlight the anti-absence role of feed-forward inhibitory PV+ interneurons of the TC-loop in non-epileptic animals.

Recently, a reversible time-controllable model of absence seizure has been developed [107]. A Tet-Off system controlling the expression of the ArchT (archaerhodopsin from the Halorubum strain) exclusively in the PV+ interneurons of the nRT was able to induce ETX-sensitive ASs in the absence of light stimulation and of doxycycline. This occurs due to the toxic effect of ArchT on the T-type calcium currents [107].

### 2.6. What Did We Learn from These Models of CAE?

From the extensive research conducted on animal models of ASs, several conclusions have begun to emerge.

First, ablating a single ion channel or regulatory protein in a specific neuronal population within an isolated structure of the TC-loop can be sufficient to induce spontaneous ETX-sensitive ASs. Importantly, genetic manipulation may trigger compensatory mechanisms in other parts of the TC- loop that may manifest in the opposite direction depending on the region of the circuitry under consideration.

Secondly, inputs to the thalamus from different brain regions including the basal ganglia, cerebellum, and brainstem can modulate SWDs by either increasing, decreasing, or suppressing their occurrence.

Thirdly, glial cells likely play an active role in both the development and progression of epilepsy. Indeed, ASs promote hypersynchrony within thalamocortical networks, leading to increased oligodendrogenesis and myelination within epileptic circuits. This, in turn, enhances circuit efficiency, further reinforcing seizure activity.

Fourthly, the phenotypic consequences of a mutation in polygenic diseases such as absence epilepsy can vary significantly depending on genetic background. Studies using congenic rat strains indicate that the dominant mutation identified in GAERS acts as a modulator of seizure expression rather than as sole driver of ASs generation.

Finally, numerous epistatic interactions (between different genes) and gene–environment interactions can disrupt the TC-loop, leading to ASs with slightly distinct characteristics. This is evident when comparing ASs phenotype in GAERS and WAG/Rij models.

## 3. Juvenile Myoclonic Epilepsy (JME)

### 3.1. Characteristics of JME in Humans

JME, also known as “Janz syndrome” or “impulsive petit mal”, is the most common IGE syndrome, affecting approximately 10% of all people with epilepsy and approximately 27% of those with IGE. The typical age at onset ranges from 10 to 24 years with a peak between 12 and 18 years. Unlike CAE, JME is generally considered a lifelong disorder, often requiring lifelong therapy, as seizure recurrence occurs in approximately 75% of cases after medication withdrawal [108]. JME is characterized by a triad of clinical manifestations whose appearance is age dependent. Myoclonic jerks (MJs) (shock-like, irregular, arrhythmic, clonic movements) must be present and mainly affect the upper extremities, occurring without loss of consciousness. On an EEG, MJs correspond to a generalized burst of fast polyspike-and-wave discharges (4–6 Hz) lasting 0.5–2 s. In over 90% of JME patients, generalized tonic–clonic seizures (GTCSs) emerge a few months/years later. Absence seizures occur in one third of JME cases but are typically less severe than in CAE, lasting 1–3 s, and occurring less than daily, displaying intra-discharge SWDs frequencies ranging from 3 to 10 Hz. MJs occur commonly in clusters within the first hour after awakening and usually precedes GTCSs. The frequency of GTCSs is variable. Sleep deprivation, fatigue, and excessive alcohol intake are the most powerful precipitants of MJs and GTCSs, while photosensitivity is also reported in approximately 30–40% of patients. The most effective AED is VPA but levetiracetam and lamotrigine are also effective options [109]. Notably, up to 30% of JME patients are refractory to anti-seizures medication.

A rare quantitative neuropathological study of JME brains demonstrated increased cell density in the frontal, temporal, and occipital lobes at all ages analyzed along with microdysgenesis, corresponding to diffuse single cell heterotopias in the stratum moleculare and in the subcortical white matter [110]. Structural MRI studies frequently report decreased GM volume in the thalamus, with some also identifying a reduction in the volume of the hippocampus or putamen (for a review see [111]). One study reported incomplete uni- or bi-lateral hippocampal inversion (IHI or HIMAL) in 50% of JME patients and their unaffected siblings [112]. Although IHI is not an etiological factor in epilepsy, it could be a sign of disturbed cerebral development leading to epilepsy since inversion is normally completed at gestational week 25 [113]. DTI studies consistently indicate differences in WM integrity in the thalamocortical network and the corpus callosum [111]. Importantly, studies of the dynamic changes occurring during the first two years of JME reveal altered brain developmental trajectories in the fronto-parieto-temporal regions [114]. Moreover, the differences in GM, WM, and in certain behavioral tests are already present at seizure onset, confirming they precede the phenotype and are not a consequence of repetitive seizures [115,116]. Modest but significant changes in some cognitive and behavioral responses are also reported together with slightly increased anxiety, depression, and mood disorder [111].

As with CAE, JME has a well-established polygenic component, though genetic studies have been relatively disappointing. One of many possible explanations could be that JME is not a “single” disease entity but comprises several subsyndromes, as proposed by the group of Delgado-Escueta [117]. The latest GWAS study from ILAE identified *GABRA2*, *RPSO2*, *TMEM74*, *STX1B*, and *CACNAI* as prioritized genes in three significant loci [17]. Once again, three of these genes are not ion channels. However, although monogenic forms of JME are much less common than polygenic ones, several genes associated with these forms of the disease have been identified [118].

### 3.2. Spontaneous Models of JME in Mammals

#### 3.2.1. Epileptic Baboons

This model was initially identified when Robert Naquet observed photosensitivity in the *Papio hamadryas papio* baboon subtype. Later, the epileptic phenotype was documented in other baboon subspecies housed at the Primate Research Center of San Antonio where 26% of baboons exhibited generalized myoclonic or tonic–clonic seizures, typically occurring in the morning [119,120]. By the last year of adolescence (i.e., six years old), over 90% of epileptic baboons experienced GTCSs [121]. Histological analysis revealed an average 37% reduction in neuronal numbers in the cortex, with the decrease being more pronounced in the frontal areas related to motor function. Additional studies are needed to determine whether these changes are a cause or a consequence of epileptic seizures [122]. Voxel-based morphometry indicates increased GM in frontopolar, orbitofrontal, and anterolateral temporal cortices but decreased GM in primary visual cortices and specific thalamic nuclei including the nRT [123]. At present, there is no correlation between MRI changes and histopathology but GM changes may reflect neurodevelopmental abnormalities potentially related to increased cellularity or decreased synaptic pruning [114].

A genetic study of the determinants underlying this epilepsy identified a significant association with a single nucleotide polymorphism (SNP) located in an intronic region of *RBFOX1* [124]. No protein-altering variant reached significance, though GSEA highlighted the enrichment of extracellular matrix structures and collagen formation genes [124]. RBFOX1 is a member of a family of brain-enriched RNA binding proteins, known to regulate alternative mRNA splicing. The neuronal deletion of *Rbfox1* renders mice susceptible to spontaneous and kainic acid-induced seizures [125]. In the hippocampus of these mice, RNA-seq analysis identified alterations in both alternative splicing and the mRNA abundance of many genes, including some encoding ion channels. Of note, Vamp1 expression, an interneuron-specific protein-regulating synaptic transmission, is strongly downregulated [126]. Interestingly, RBFOX1-mediated splicing in interneurons appears to be cell-type specific, further complicating studies on the consequences of its inactivation [127]. These findings strongly support the notion that RBFOX1 may also be implicated in the pathophysiology of the baboon seizures. However, the functional consequences of the SNP on the expression of RBFOX1 remains undetermined. This information is crucial for understanding the physiopathology of JME in this species. Indeed, even a highly significant non-coding SNP may exert functional effects on another gene, even at a distance of megabases [128].

#### 3.2.2. Epileptic Dog Model

Epilepsy is the most common chronic neurological disorder in dogs and genetic studies have identified multiple causative genes. For example, some Rhodesian Ridgeback (RR) dogs develop a unique epileptic phenotype with an onset at a mean age of six months, characterized by frequent myoclonic jerk. On average, within six months, 38% of affected dogs also develop GTCSs [129]. Generalized myoclonic seizures are photosensitive and can be triggered by everyday visual stimuli, with levetiracetam and potassium bromide reported to be the most effective AED [129]. Genetic analyses have identified a homozygous 4bp deletion in the *DIRAS1* gene, a member of the Ras family of small GTPases. This frameshift mutation alters the last 10 amino acids of the protein, introducing 104 extra amino acids. Although this does not affect the functionality of the RAS domain, it likely renders the mutated protein functionally altered, as suggested by the more diffuse soma staining observed in neurons from RR dogs [129]. In mammals, the biological functions of DIRAS1 are not well understood, but its ortholog regulates synaptic activity (i.e., acetylcholine release) at the neuromuscular junction of *C. elegans*, while influencing cell migration, neurite outgrowth, and dendrite architecture in the developing nervous system of zebrafish [130,131].

### 3.3. Models of JME Based on Genetic Manipulation of Mendelian Genes

Historically, genetic studies have identified 29 chromosomal loci linked to the Mendelian forms of JME, but the variants responsible for the disease have only been identified for a few of them. Although these monogenic forms of JME are much less frequent, they can be used to develop invaluable models that will help to elucidate the pathophysiology of the disease. This section will focus on describing the data that has been reported for JME.

#### 3.3.1. GABRA1

In 2002, the first mutation associated with JME was identified in the *GABRA1* gene of a large French-Canadian family. This autosomal dominant mutation was fully penetrant and consists of an Ala322Asp (A322D) missense mutation, which affects a conserved residue in the third transmembrane domain of the α1 subunit [132].

When co-expressed in heterologous cells along with ß1 and γ2 subunits, the A322D mutation decreases the GABA inhibitory current, due to its rapid proteasomal degradation and its interference with the trafficking and cell surface expression of the other two subunits. When overexpressed in cultured cortical neurons, the mutated subunit reduces the amplitude and time course of miniature inhibitory post-synaptic currents (mIPSCs) by decreasing the surface expression of the α3ß2γ2 receptors. This suggests a haplo-insufficient and dominant-negative effect of the A322D mutation [133,134]. *Gabra1* KO and HET mice experienced ETX-sensitive absence-like seizures in two different genetic backgrounds, with the absence of *Gabra1* leading to a significant reduction in viability from P30 onwards [135]. A knock-in (KI) mouse line carrying one A322D mutated allele (*Gabra1* Het-KI) also experiences absence seizures from P35 onwards [136]. While age does not affect the incidence of SWDs, both *Gabra1* Het-KI and *Gabra1* HET exhibit spontaneous myoclonic seizures and polyspike discharges at P120, although these occur less frequently than SWDs [136]. Molecular analysis revealed that for both mice, the ß2/ß3 and γ2 subunits expression is not affected (suggesting the total expression of GABA receptors remains constant) whereas α3 subunit expression is increased in the frontal cortex, with no differences between the lower or upper layers of the motor and somatosensory cortex. The density of gephyrin clusters in layer 2/3 of the motor cortex, M1, is not affected. Finally, for both mutants, the peak amplitude of mIPSCs is decreased and the decay constant is prolonged in layer 2/3 of the M1. Altogether, these changes are expected to increase neuronal excitability and synchrony, but do not fully explain the worsened phenotype observed at P120 [136]. More recently, a zebrafish mutant line lacking *gabra1* was developed resulting in premature death (>90% death by 10 weeks) [137]. The *gabra1* knockout larvae are hypoactive but undergo intense seizures immediately after the lights are turned on. Interestingly, a one-day incubation with VPA or clonazepam completely inhibited the seizures, whereas levetiracetam and carbamazepine only partially rescued them. No major qualitative differences in neuronal fibers or neuronal content could be observed in the mutant line. However, RNA-seq analysis revealed an alteration in many genes involved in axon guidance, axonal branching, synaptic docking, and endocytosis. This indicates that although the development of the inhibitory cell population is not affected, GABRA1 plays a key role in the establishment of complex branching within the inhibitory network of the brain [137].

#### 3.3.2. EFHC1

*EFHC1* (EF-Hand containing-1) is a gene located on chromosome 6p12, a susceptibility locus long associated with JME, and was the second gene identified to exhibit Mendelian transmission. It was first found to be mutated in 21 affected members of 6 unrelated families from Belize and Mexico [138]. Following this discovery, several new mutations were identified in many other patients from Mexico, Honduras, Japan, Italy, Austria, Pakistan, and India. However, screenings of patients from Germany, The Netherlands, Sweden, and the United Kingdom found these mutations to be extremely rare (for a review see [111]). The mode of inheritance is autosomal dominant with incomplete penetrance and almost all identified mutations are heterozygous missense mutations [139]. In a few cases, the mutation was located in the 3′UTR, and in one report the mutation induced a frameshift affecting the shorter isoform of the protein. EFHC1 contains three DM10 and one putative EF-hand domain. The residues affected by missense mutations are distributed along the protein including the DM10 domains, the regions between them, the putative EF-hand, and in the C-terminal region [140].

This gene remains one of the most enigmatic as it encodes for a protein whose exact cellular function is largely unknown. Consequently, determining the results of mutations rely primarily on in silico prediction algorithms, which often provide heterogeneous results. However, by applying NHGRI guidelines and ACMG combinatorial criteria, 9 variants were classified as “pathogenic”, 14 as “likely pathogenic”, 20 of “unknown significance”, 8 as “benign”, and 3 as “likely benign” [140].

Regarding the functions of EFHC1, it has long been known that Rib72, its *Chlamydomonas* orthologue, localizes to the flagella and, along with tubulins and tektins, is one of the main components of the stable protofilament ribbon [141]. Subsequent research has established that EFHC1 and its orthologue are highly expressed in cells with motile cilia, such as *Tetrahymena* and *Chlamydomonas*, as well as in mouse tissues rich in motile cilia, such as the testis, lung, kidney, and brain [142]. When EFHC1/RIB72 is inactivated in these organisms, a reduction in the beating frequency of motile cilia is observed, without any changes in their length or structure [143,144,145]. Consistent with this observation, hydrocephaly was reported in *Efhc1*-KO mice but not in *Efhc1*-HET mice [145]. In *C. elegans*, a nematode containing 302 neurons and only non-motile sensory cilia, the EFHC1 orthologue (which lacks the putative EF-hand and contains only two DM10 domains) is expressed in a subset (12 in total) of ciliated mechanosensory neurons, i.e., the dopaminergic CEP, ADE, and PDE neurons and the glutamatergic OLQ neurons [146]. In dopaminergic neurons, EFHC1 localizes to both the cilia and the synapse. At the synapse, EFHC1 partially overlaps with the active zone protein ELKS1 and likely modulates dopamine release, as suggested by the behavioral tests performed on loss-of-function *efhc-1* mutants [146]. In *Drosophila*, the *Defhc1.1*-KO fly exhibit overgrowth of the dendritic arbor of class IV neurons (i.e., the non-ciliated neurons acting as nociceptors), and an increased number of satellite boutons at the neuromuscular junction, leading to increased spontaneous neurotransmitter release [147]. In stable boutons, Futsch (the ortholog of MAP1B) is associated with loops of bundled MTs, but in KO flies, most boutons lack these Futsch-positive loops, suggesting that Defhc1.1 is a negative regulator of MT dynamics [147]. These defects are reversed by the specific neuronal overexpression of *Defhc1.1*, confirming that they result from *Defhc1.1* loss of function in the nervous system [147]. In EFHC1b morphants of *X. laevis* embryos, axoneme formation in ciliated cells is inhibited and morphants display defects in the central nervous system and neural crest patterning, producing noticeable effects on their morphology [148]. The authors suggest that if these effects occur in response to EFHC1 mutations during human brain development, they could affect brain organization and function [148].

Experiments involving the ectopic overexpression of EFHC1 or its mutated forms have been conducted to attempt to better understand its cellular functions. The first functional test was performed on primary cultures of hippocampal neurons and showed that the overexpression of EFHC1 induced R-type calcium channel-dependent apoptosis, which was reduced when mutated forms of EFHC1 were overexpressed [138]. The authors concluded that EFHC1 mutations compromised its apoptotic activity, preventing the elimination of unwanted neurons during brain development [138]. Other experiments showed that EFHC1 associates with mitotic spindles, the midbody, and the centrosome when overexpressed in cell lines [149]. The overexpression of mutated EFHC1, but not polymorphisms variants, led to abnormal mitotic spindles and impaired cell division. This finding was replicated for 12 of the 13 EFHC1 mutants discovered in India [150,151]. Biochemical analysis confirmed that EFHC1 physically interacts with tubulin [150]. In mice, in utero *electroporation* (IUE) experiments demonstrated that knockdown or overexpression of pathological mutants impair but do not block (as some authors have speculated) the radial migration of cortical projection neurons and the tangential migration of interneurons [150,152]. Indeed, the neuronal progenitors of cortical projection neurons remain in the “multipolar” transition state for longer, but do finally reach their assigned cortical layer; however, their dendritic arbor is less developed and the axonal growth towards the contralateral region is delayed [153].

Considering these findings, one can wonder about the cellular role(s) of EFHC1. Although it may be a multi-functional protein, several studies converge on at least one identical interacting partner in different species: tubulin. Indeed, human EFHC1 directly interacts with MTs in vitro and, when overexpressed in cell lines, it associates with tubulin-rich structures such as the mitotic spindles, centrosome, and midbody [149,150,151]. *Drosophila*, *Defhc1.1* interacts directly with MTs in vitro, associates with the mitotic spindles, and colocalizes with MTs in axons and synaptic boutons [147]. Finally, CryoEM analysis of *Chlamydomonas* and *Tetrahymena* flagella/cilia further detailed the RIB72–tubulin interaction, showing that RIB72 acts as a microtubule inner protein (MIP) [143,144,154]. Note that the acetylation of K40 residue of the α-loop is not required for the interaction with DM10 domains [155].

Despite this biochemical and structural evidence, key questions remain. First, the effects of EFHC1/RIB72 mutations on MT binding are unknown. In silico modeling suggests that mutations in the DM10 domain alters its secondary structure and interaction capability, but not all mutations are located in these domains [147,151]. Second, the apparent specificity of EFHC1 for some flagellar protofilaments remains unexplained. Finally, the impact of EFHC1/RIB72 binding on MT properties and dynamics require further investigations. Results from *Drosophila* suggest an influence on MT dynamic in vivo; however, EFHC1 does not affect MT growth rate in vitro, nor does its absence alter cilia or flagella structure although proteomic studies revealed changes in the MIP repertoire [145,150,156].

Despite these questions, we believe that the many observations reported above, including those on ectopic overexpression, may at least partially be explained by altered MT properties/dynamics as suggested for *Defhc1.1*. Indeed, cryo-ET revealed that the lumen of the cytoplasmic MTs of neurons and other cell types is not a hollow tube but is occupied by a range of morphologically diverse components or MIPs. Interestingly, the abundance of these luminal particles increases with neuronal differentiation, but also correlates with MT curvature, lattice defects, and freshly polymerized plus ends [157]. Some of these luminal particles have been identified as MAP6, αTAT1 (the enzyme that acetylates the K40 residue of α-tubulin), or F-actin among others [158]. Note that Tau has binding sites inside or outside the MTs depending on whether it is added before or after MT polymerization [159]. Much remains to be discovered about these MIPs, but it is speculated that their binding to cytoplasmic MTs may be a mechanism for modulating MT dynamics without restricting the binding of cargos (among others) located on the outer surface or may be a mechanism for localized MT repair.

Regarding the epileptic phenotype, both *Efhc1*-KO and *Efhc1*-HET mice exhibit spontaneous myoclonus and increased sensitivity to PTZ-induced seizures [145]. The mechanism behind this sensitivity remains unknown but is unlikely to be solely related to cilia beating frequency, as this parameter remains unchanged in adult *Efhc1*-HET mice [145]. One hypothesis could be that EFHC1 affects the choroid plexus (CP) as it is expressed in this structure during development [160]. During embryogenesis, the cerebrospinal fluid (CSF) contains molecules critical for brain development and specifications including SHH, LIF, FGF2, IGF2, or Wnts (for a review [161]). These morphogens are released from the CP by apocrine secretion and exocytosis, two MT-dependent mechanisms that both have obvious effects on brain development [162,163,164]. Alternatively, EFHC1 mutations may impact the glymphatic system, which is implicated in the metabolic and soluble protein clearance from brain interstitial fluid [165]. Interestingly, this system is sensitive to conditions that favor seizures, such as alcohol and sleep, and is less efficient in JME patients [166,167,168]. Therefore, EFHC1 mutations could impair the appropriate management of environmental conditions that favor seizures onset. The resultant effect is epileptic seizures in the context of a “sensitized brain” but would be without detrimental effect on a typically developing brain, explaining why this gene appears “tolerant” to mutations.

#### 3.3.3. GABRD

GABRD, the gene encoding the δ subunit of the GABA_A_ receptor, was initially reported as mutated in one GEFS+ and one JME family [169]. The R220H mutation found in JME patients is autosomal dominant, and results in a decreased surface expression of the receptor, while channel gating is also altered [170]. The δ subunit-containing GABA_A_ receptors are predominantly located in extra or peri-synaptic positions, where they mediate a slow constant inhibitory current known as tonic inhibition [171]. These GABA_A_ receptor types are highly expressed in the thalamus, and their activation in the VB promotes a shift in cell firing from a tonic to a bursting mode [172]. *Gabrd*-KO mice are more prone to PTZ-induced seizures but specific deletion of the subunit in interneurons renders the mice resistant to kainic acid-induced seizures [173,174]. However, no replication of GABRD mutations in JME patients have been reported since 2004, but a more recent study identified several gain-of-function variants in GABRD in patients with neurodevelopmental disorders and epilepsy [175].

#### 3.3.4. CASR

Several missense variants of the *CASR* gene, encoding the G-protein-coupled Calcium Sensing Receptor, co-segregated in affected members of a three-generation family and in five individuals from South India have been detected [176]. Recently, a heterozygous nonsense mutation was found in a child with idiopathic epilepsy and autism [177]. One reported mutation, located in an arginine-rich motif in the c-terminus, increases the receptor’s abundance at the plasma membrane, thereby enhancing its activation of intracellular signaling [178]. *Casr*-KO mice suffer from developmental brain delays, and the expression of several neuronal and glial differentiation markers is decreased in this model [179]. Although the proliferation of neural stem cells is not affected, their differentiation capacity is diminished [179]. Interestingly, CaSR is considered as a marker of newly born oligodendrocytes (OLs) and has been shown to impact myelin formation [180,181]. CaSR also modulates neurite extension and branching in sympathetic and hippocampal pyramidal neurons [182]. Finally, it modulates synaptic transmission by controlling the Na^+^-leak channel non-selective (NALCN) and the presynaptic non-selective cation channel (NSCC) [180].

#### 3.3.5. BRD2

EJM3 is a major JME susceptibility locus, mapped to chromosome 6p21 through independent linkage studies. An autosomal dominant mutation (with incomplete penetrance) was identified as an SNP in the promoter region of the *BRD2* (bromodomain containing 2) gene [183]. This SNP is located in a CpG island, a region of DNA sensitive to methylation, suggesting it may affect gene expression. Therefore, the methylation status of the promoter was tested and found to be highly methylated (meaning *BRD2* is silent) in lymphoblastoid cells from JME patients of Caucasian origin [184]. However, this discovery was not replicated in a German population by another group, although it should be noted that the SNP showed no association with JME in this population [185,186]. Despite this, the concept that the heritability of JME could be partly epigenetic was groundbreaking and indeed merits further investigation, particularly because many SNP may appear “irrelevant” due to their lack of effect on protein sequences.

The link between BRD2 and JME is strongly supported by mouse models. BRD2, also known as RING3, is a transcriptional regulator that may associate with both transcription complexes and acetylated chromatin via acetylated lysine-12 residue of histone H4 [187]. *Brd2*-KO mice begin to die at E9.5, are notably smaller, and exhibit neural tube abnormalities indicative of defective closure, suggesting *Brd2* is essential for neural development [188]. Indeed, cell death is increased in the *Brd2*-KO embryo and mouse embryonic fibroblasts (MEFs) show compromised proliferation due to a delay in the G1 phase of the cell cycle. *Brd2*-HET MEFs proliferate at an intermediate rate, but *Brd2*-HET mice are viable and overtly normal [188]. Despite this, more than half of *Brd2*-HET females exhibited spontaneous seizures [189]; moreover, both male and female mutant mice showed increased sensitivity to flurothyl-induced seizures [189]. Brain analysis reveals that the number of GABAergic neurons (GAD67+) is significantly decreased in structures involved in controlling seizure activity such as the neocortex, striatum, thalamus, and substantia nigra reticulata [189]. A more detailed analysis found that changes in the GABAergic system precede the onset of seizure susceptibility. Specifically, at P15, a decrease in PV+ neurons number is observed in structures such as the striatum and the M1 neocortex, whereas flurothyl-induced seizure susceptibility begins around P30. Moreover, at P15, the PV+ neurons of *Brd2*-HET mice show reduced dendritic arborization and smaller neuronal bodies, two morphological features of immature neurons [190]. In zebrafish, knockdown of either Brd2a or Brd2b results in excessive cell death and dysmorphology of the CNS, exemplified by a reduced hindbrain volume and an ill-defined midbrain–hindbrain boundary, confirming the essential role of BRD2 during CNS development [191,192].

#### 3.3.6. CILK1/ICK

The last gene identified by linkage and whole exome sequencing is *ICK* (intestinal cell kinase), now renamed *CILK1* (ciliogenesis associated kinase 1). Autosomal-dominant missense mutations co-segregate with the affected members of two large and eight medium-sized JME families from Mexico, Honduras, Belize, and Japan. In this study, a total of 21 pathogenic variants in 22 additional patients were identified [193]. Notably, homozygous mutations in the same gene, though affecting different residues, are reported in neonatal lethal syndrome of endocrine-cerebro-osteodysplasia (ECO syndrome) and in some patients with short rib polydactyly syndrome (SRPS) [194,195].

CILK1 is a Ser/Thr-kinase belonging to the CMGC family: the catalytic domain is located in the first half of the protein, followed by a nuclear localization signal, while the second half of the protein exhibits the characteristics of an intrinsically disordered domain with no homology to other proteins [196]. The kinase activity is regulated by CCRK (cell cycle-related kinase or CDK20) and PP5 (protein phosphatase 5 or PPP5C), both of which target the Thr157 residue of the TDY motif [197]. Recently, the FGFR3 receptor was found to interact with CILK1 to phosphorylate Tyr15, interfering with optimal ATP binding and partially inhibiting the kinase activity [198]. To date, four substrates of CILK1 have been identified: KIF3A, Scythe (BAG6), Raptor, and GSK3ß [197,199,200,201]. A protein named SDCCAG8, which is localized to the centrosome/basal body and is essential for primary cilia formation, was also found to interact with CILK1 [202].

When overexpressed, CILK1 is localized in the primary cilia of cells but is also observed in other cellular compartments including the cytoplasm and nucleus. Mutation of the Arg272 residue found in ECO syndrome is sufficient for the protein to be excluded from the nucleus, whereas the non-catalytic c-terminal domain is required for cilia localization [200,203]. In mice, the deletion of *Cilk1* is lethal, with the mice dying around birth, likely due to respiratory failure. *Cilk1*-KO displays an enlarged cerebral cortex, cleft palate, smaller lung lobes, polydactyly, and delayed skeletal development [204,205]. At E15.5, only a few neural progenitors in the cerebral cortex possess a cilium, although dorsal–ventral neural patterning is not significantly affected [204]. In the absence of CILK1, the localization of Shh pathway components is disrupted, affecting the signaling cascade [204,205]. Interestingly, in nestin-cre cKO mice at P30, both the cerebellum and dentate gyrus (DG) are smaller, but the primary cilium appears normal [204].

Importantly, IUE experiments demonstrated that overexpression of pathological mutated forms of CILK1 (both from ECO and JME) impaired the division of cortical progenitors of the dorsal telencephalon and hindered the radial migration of neuroblasts [193]. Since mutated residues reported in JME are dispersed along the protein length and are not highly conserved, their relevance to the properties of CILK1 has been challenged [196]. A functional test revealed that JME mutations located in the kinase domain abolished kinase activity, while those in the c-terminal domain affected CILK1’s ability to regulate cilia length and promoted ciliogenesis [206]. In accordance with these observations, a transposon-mediated somatic mutagenesis screen demonstrated that the inactivation of a single Cilk1 allele affected the migration of neuronal precursors to the cortex, a result confirmed by shRNA knockdown [207].

Although no spontaneous seizures were recorded in *Cilk1*-HET mice, they were more prone to tonic–clonic seizures and polyspikes under isoflurane anesthesia [193]. This suggests that, although the brains of *Cilk1*-HET mice do not show gross brain abnormalities, more subtle changes may be present as demonstrated with *Brd2*-HET mice (see above).

### 3.4. Models of JME Based on Genetic Manipulation of Other Mutated Genes

Unlike CAE, where the neuronal circuitry responsible for seizures is well defined, no clear mechanistic hypothesis has been proposed or tested for JME.

In addition to the canonical Mendelian genes, several studies have identified mutations in genetic loci not necessarily located in previously reported JME cases. Below, we will briefly describe some examples.

SNP and missense mutations have been reported in the gene encoding carboxypeptidase A6 (CPA6) in patients with temporal lobe epilepsy, febrile seizures, and JME [208]. The two mutations found in JME patients resulted in reduced enzyme activity or protein level in the extracellular matrix [208]. No knockout mice have been produced to date, but knockdown of *cpa6* in the zebrafish results in resistance to the effects of PTZ and pilocarpine on swimming behavior [209].

A study reported an association between two TAP-1 missense mutations and the most common IGE in the Tunisian population [210]. The protein affected was an ATP-binding cassette transporter and analysis of the double ß2m/TPA1-KO demonstrated reduced MHC class I surface levels, leading to altered homeostatic regulation of synaptic function and morphology during development [211].

Very recently, a GWAS for impulsivity in JME patients revealed that an SNP in the intergenic region, near *SLCO5A1*, decreases its expression in the GTEx cerebral cortex [212]. The *SLCO5A1* gene encodes a membrane-bound organic anion transporter (its substrate remains unknown) and is highly expressed during human brain development. In flies, knockdown of *Oatp308*, the closest homologue of SLCO5A1, induced striking over-reaction to vibration stimuli and a dramatic increase in seizure-like events induced by hyperthermia [212].

## 4. Generalized Tonic–Clonic Seizures Alone (GTCSA)

### 4.1. Characteristics of GTCSA in Humans

GTCSA accounts for approximately one third of adolescent IGE and onset is typically between the ages of 10 and 25 years. Although relatively infrequent, seizures usually occur within 2 h of awakening and are sometimes triggered by sleep deprivation, fatigue, or alcohol intake [213]. Many patients require medication throughout their adult life and the preferred AED are VPA, lamotrigine, levetiracetam, topiramate, perampanel (an AMPAR antagonist), and zonisamide [214]. An MRI study focusing exclusively on patients with GTCSA revealed bilateral thalamic atrophy and cortical thinning most prominent in fronto-central areas [215]. In drug-resistant patients, atrophy of the basolateral left amygdala has also been reported [216]. Although intelligence is within normal range, some specific cognitive abilities, such as attention or decision making are sometimes altered [217].

Up to now, no specific gene has been associated with GTCSA and the latest GWAS study from ILAE did not find any significant loci for this form of IGE [17].

### 4.2. Spontaneous Models of GTCSA in Mammals

#### NER Model

The Noda epileptic rat (NER) is derived from brother–sister breeding of three females and one male from Crj:Wistar rats at Charles River Japan: these rats exhibit spontaneous GTCSs. At 2 months of age, NERs first exhibit abnormal behaviors that are not yet classified as GTCSs. Then, around 14 weeks of age, 94–98% NERs experience spontaneous GTCSs with a frequency of about one every 30 h [218]. Neither tactile, photic, nor acoustic stimuli induce GTCSs, but these rats are twice as susceptible to PTZ-induced seizures. Tossing-stimulating conditions provoke GTCSs, beginning at 5 weeks of age at the earliest, with occurrence increasing up to 90% by 15 weeks of age [218]. Adult NERs are also susceptible to environmental disruptions such as bedding replacement or unpleasant sensory stimuli, but seizures are sensitive in a dose-dependent manner to phenobarbital and VPA [219]. Two seizure susceptibility loci have been mapped on rat chromosomes 1 (*Ner1*) and 3 (*Ner2*). It is speculated that *Ner1*, an autosomal recessive locus, controls the inheritance of spontaneous seizures; while *Ner2* affects the occurrence of tossing-induced seizures [220]. A second study covering all rat chromosomes identified a third GTCS-associated locus, *Ner3*, which genetically interacts with *Ner1* for the development of GTCSs [221]. Global gene expression analysis revealed that *Cckbr* (Cholecystokinin B receptor) and *St5* (suppressor of tumorigenicity 5), located in Ner1, and *Phf24* (PHD finger protein), which maps to *Ner3*, are downregulated [221]. The decreased expression of PHF24 results from retrovirus insertion in the second gene intron [221].

The GTCSs are evoked primarily by the activation of the limbic and/or cortical circuits, as demonstrated by c-fos labeling of the cerebral cortex (prefrontal, piriform, peri-entorhinal, insular, motor, sensory, and auditory cortex), and certain limbic areas (amygdala, CA3, and DG of the hippocampus), while basal ganglia, diencephalon, and lower brainstem structures remains unaffected [222]. By measuring total amino acid concentration in different brain regions, variations were detected only in the cerebellum, specifically for glycine and taurine [223]. At 8 weeks, an increased NPY (a supposed anticonvulsive peptide) content was reported in the amygdala and the striatum. By 16 weeks of age, when GTCSs are expressed, NPY content was elevated in the frontal cortex, the striatum, and some limbic regions (hippocampus, entorhinal, and piriform cortex) [224]. In the hippocampus, this was accompanied by a change in NPY2R and NPY5R expression [225]. Furthermore, CA3 neurons exhibit abnormal epileptiform discharges possibly due to the dysfunction of T-types calcium channels [226]. A decreased expression of Kir4.1 (inwardly rectifying potassium channel subunit) has been observed in astrocytes of the occipito-temporal region and thalamus [227]. In the amygdala nuclei that contributed to limbic hyperexcitation, Kir4.1 expression was selectively diminished in the astrocytic processes, while it remained unchanged in the somata [227]. This finding is notable because repeated treatment with AEDs, such as VPA, phenytoin, or phenobarbital (but not ETX), have been shown to elevate Kir4.1 expression in the cerebral cortex, the amygdala, and the hippocampus [228]. Finally, PHF24, also known as GINIP (Gα inhibitory interacting protein) has been shown to bias GPCR responses in favor of Gβγ over Gαi signaling. Given its cellular function, GINIP may affect any Gαi-coupled GPCRs in both inhibitory and excitatory neurons. GINIP is essential for preventing imbalances in neurotransmission underlying seizure susceptibility, as demonstrated by the increased sensitivity of GINIP-KO mice to bicuculline-induced seizures [229].

## 5. Participation of the Thalamus in JME and GTCA Seizures

Although genetic models of JME and GTCA have not been as thoroughly characterized as those for absence epilepsy, they may provide important insights regarding the difference in pathogenic mechanisms between absence and convulsive seizures. Indeed, for both epilepsy types, numerous brain imaging analyses in humans, or sometimes invasive studies in rodent, demonstrate the involvement of distinct thalamic nuclei in various seizure types (for a review see [230]).

Traditionally, being a component of the TC-loop controlling ASs, the nRt is considered to be a diffusely organized structure responsible for inhibiting thalamic nuclei (see Section 2.2). However, evidence suggests that the nRT is divided into distinct regions (limbic, motor, and sensory for example), each connected to multiple thalamic nucleus and cortical areas [231]. For instance, the nRT receives input from the hippocampus and projects back to it via the reuniens nucleus of the thalamus, forming “thalamo–hippocampal” connections that resemble the circuit linking the sensory thalamus to the cortex [232]. High-frequency stimulation of the nRT increases both the latency for the development of PTZ-induced GTCSs and the number of PTZ injections required to induce status epilepticus [233]. Additionally, the optogenetic stimulation of PV+ interneurons of the nRT, at high frequency and long duration, significantly reduced the seizures induced by 4-aminopyramine injections [234]. Interestingly, PV+ and Somatostatin+ (SOM+) interneurons have distinct electrophysiological properties, are located in different regions of the nRT, and participate in largely non-overlapping anatomical circuits. Indeed, PV+ cells are primarily involved in sensory circuits, while SOM+ cells are more associated with non-specific thalamocortical relay nuclei that project to various cortical regions, including the prefrontal cortex [235,236]. The anterior thalamic nucleus (ANT) is a higher-order thalamic nucleus that indirectly connects to the limbic system and contributes to reciprocal hippocampal–prefrontal interactions. Early studies performed on guinea pigs showed that muscimol (a GABA agonist) injections into the ANT confer protection against PTZ-induced seizures [237]. Finally, genetic models of atypical absence seizures have been obtained by overexpressing either GABBR1 or GABBR2 in the forebrain. SWDs were recorded in the midline thalamus, hippocampal CA1 region, and nRT, supporting a role for the limbic pathway in generalized epilepsies [238].

## 6. Non-Mammalian In Vivo Models

While most research on IGE has been conducted in rodents, other animal models have emerged over the last decades, and have been cited in the text when used to study specific genes mutated in IGE.

One such model is the fruit fly *Drosophila melanogaster*. Despite a genome approximately 25 times smaller than that of humans, it contains around 13,700 protein-coding genes, which is about half of the human coding genes [239]. Importantly, about 75% of human disease-related genes have orthologues in the fly, making it an attractive alternative or complement to mouse research. Advantages of *Drosophila* include low maintenance cost, minimal ethical restrictions, and access to a powerful genetic toolbox which includes the latest technologies such as optogenetic and light-inducible gene expression [240]. Throughout the last decades, substantial progress has been made in studying epilepsy in flies. Seizure-like activity can be induced by direct brain electrostimulation, mechanical disturbances, or temperature elevation [241]. In contrast, chemically induced seizures are rarely used in this model. To demonstrate the relative utility of *Drosophila* as a model, one study showed that optogenetic modulation of neuronal activity during a critical embryonic window (egg laying stage) was sufficient to change seizure-like behavior in third instar larvae [242]. Indeed, increasing neuronal excitation in WT Drosophila during this period permanently induced seizure behavior. Conversely, optogenetic silencing during the same period suppressed seizure activity in a seizure-prone mutant strain, the bang-sensitive mutant [242].

Another emerging model is the zebrafish (*Danio rerio*), which is phylogenetically closer to rodents and humans than the fruit fly. The zebrafish genome comprises 25 chromosomes pairs and approximately 26,000 protein-coding genes. Moreover, nearly 84% of human disease-associated genes have an orthologue in zebrafish [243]. Due to genome duplication events during teleost evolution, zebrafish often carry duplicated genes, providing both an advantage (i.e., the sub-functionalization of pleiotropic phenotype) and a disadvantage (i.e., genetic redundancy that complicates phenotype interpretation). This model offers several practical advantages such as simple breeding and high fecundity, rapid external development, transparency at the larval stage, and efficient genetic manipulation to facilitate gene editing or knockdown during developmental stages. Emerging tools have allowed whole-brain imaging in freely moving zebrafish [244] and seizures can be detected using adapted EEG developed for both larval and adult zebrafish, visualized in larvae using transgenic reporters or inferred from motor behavior tracking [245]. Epileptiform activity can be triggered in the zebrafish by photic stimuli, stress (e.g., tank cleaning, introduction of a net into the tank), and chemical means [137,246].

Despite their advantages, limitations remain with non-mammalian models. For example, among the 145 known human “epilepsy” genes, approximatively 20% lack a clear orthologue in *Drosophila* and in 44% of the cases where there is an orthologue, mutation in the fly does not provoke epilepsy [240]. This is not entirely surprising as the adult fly brain comprises only around 100,000 neurons involved in diverse circuits supporting complex behaviors (reproduction, sleep, circadian rhythms, learning, and memory), but also because their brain structures differ considerably from those of mammals. Moreover, glial cells, while present, represent only 10% of the cells in the fly’s nervous system, far fewer than in the human brain [247,248]. Finally, the developmental processes that govern fruit fly embryogenesis also differ significantly from those in mammals. In contrast, the adult zebrafish possesses approximately 10 million neurons and shares the 4 major cell types of the human brain. Although their brain morphology more closely resembles that of mammals than the fly, the cortical architecture is very simplified and structures like the pons or cortico-thalamic and cortico-spinal tracts are absent [249]. Nevertheless, the zebrafish has a behavioral repertoire (such as anxiety, depression, reward, eating disorders, sleep, or pain) that is closer to mammalian models [250,251].

However, the predictive validity of both models for epilepsy research may be limited by a still-incomplete understanding of the mechanistic similarities between *Drosophila* or zebrafish and the human brain, particularly regarding circuitry imbalances. Furthermore, these models do not allow for the distinction of the different types of epilepsy seen in mammals. Despite these limitations, both species remain valuable for investigating neuronal functions due to the conservation of several fundamental cellular structures processes and functions across evolution. In that sense, flies and zebrafish are particularly useful for large-scale screening aiming to investigate the cellular and molecular consequences of human epilepsy-associated mutations.

## 7. Discussion

In conclusion, over the past 40 years, significant progress has been made in our understanding of the pathophysiological mechanisms underlying epilepsy, particularly in relation to absence epilepsy within the spectrum of GGE.

This progress has been driven by unprecedented development in molecular biology, electrophysiology, imaging, and histology techniques, as well as by the development of animal models, mainly rodents, with either spontaneous or genetically induced epilepsy.

A ‘simplification’ of the human situation is inherent to any (biological) model, and it is important to define the criteria a model must meet to be considered as valid. In a review of the GAERS rat model, Depaulis outlined the essential characteristics a reliable model of absence epilepsy should exhibit from a clinician’s perspective. The top criteria were, first, a specific EEG pattern of SWDs (84%), secondly, pharmacological sensitivity (48%), and thirdly, the behavioral phenotypes and the brain structures involved (46%) [252].

From this perspective, spontaneous polygenic models appear to be the closest to human pathologies. In the case of both GAERS and WAG/Rij models of ASs, many features of human absence epilepsy are apparent, including the abrupt onset and termination of SWDs, behavioral arrest during seizures and a quiet wakefulness state that favors longer seizures. These models also show high predictability for AED response because their sensitivity profile mirrors that observed in CAE patients. Furthermore, the depressive-like symptoms and cognitive deficits observed in these models are like those seen in patients [253]. Nevertheless, all rodent models differ from CAE in two aspects. First, the SWDs frequency is 5–10 Hz in rodent (except in the *swe* mouse) compared with 3–4 Hz in human. However, it is not the only example of differences in neuronal oscillations frequencies between mice and primates [252]. Second, and perhaps most importantly, ASs in rodent model do not spontaneously resolve with age (except for the few *swe* mice that survive until adulthood, see above), which limits the study of mechanisms of remission in CAE patients.

Currently, there is no widely recognized genetic rodent model that closely reproduces human syndromes for other IGE. The baboon model of Juvenile Myoclonic Epilepsy (JME) is intriguing but faces ethical and practical constraints which limit its utility in fundamental research. Regarding GTCSA, the NER model presents interesting characteristics such as an absence of induced GTCSs following simple touch or light and acoustic stimulation. Moreover, GTCSs appear spontaneously in rats in early adolescence and there are no reports of frequent and unexpected death, suggesting that this is not a major concern [218]. Additionally, the response of NER rats to AEDs mirrors that of GTCSA patients.

While GAERS, WAG/Rij, and NER rats have provided valuable insights, these strains are now inbred strains with a fixed set of mutations that are no longer “spontaneous” in nature. This highlights the ongoing need for the development of new models that incorporate the latest findings, particularly those resulting from the genetic analysis of human IGE. Indeed, most mutations identified in the coding regions are non-synonymous SNPs rather than nonsense SNPs. This distinction is important when assessing the significance of mutations in terms of the presenting phenotypes, as genes and their products function as part of the complex networks interacting both physically and biochemically within cells. This concept of “edgotype”, the relationship between genotype and phenotype through interactions, is critical. In most cases, a protein interacts with several others, and it is the sum of these interactions that contributes to the phenotype. Therefore, for the same affected gene, differences in phenotype can arise depending on whether the mutation disrupts all of the possible interactions of the protein (node suppression) or affects only specific interactions (edgetic perturbation) [254]. While knockout models have been invaluable for identifying key regulators of epileptogenesis, future efforts should prioritize more realistic IGE models such as conditional humanized knock-in models. These should be encouraged wherever possible as they allow for the expression of a mutated protein in a specific and time-controlled manner, allowing us to determine the cell-specific contribution to the phenotype. Moreover, the genetic basis of IGEs demonstrates the prevalence of complex associations between genotype and phenotype, necessitating the inclusion of the epistatic influence of other genetic variations, which itself can correspond to natural variations in gene expression level [255]. One way to tackle this question is to study the consequences of the same mutation in different genetic backgrounds in mice [41,256]. On the other hand, it should be noted that the majority of SNPs identified by GWAS studies are located in intergenic or intronic regions. More detailed analysis revealed a significant enrichment in H3K4me1 markers that are associated with enhanced transcription in dorsolateral prefrontal cortex. This suggests that the epigenetic regulation of gene expression is a potential pathophysiological mechanism contributing to the epileptic phenotype [16]. This observation takes on even more meaning when one remembers that VPA inhibits HADAC and so is an epigenetic agent [257]. This is of great importance, and we believe that epigenetic regulation should be more closely studied and considered when studying IGE.

In any case, a deeper understanding of the physiopathology of IGEs requires the elucidation of the process of epileptogenesis. Indeed, rodent models of CAE have revealed that numerous subtle brain changes precede seizure onset, suggesting potential window(s) for treatment before clinical manifestations. However, in genetic epilepsies, the latent period may overlap with different critical stages of brain development. Therefore, protein expression profiles must be analyzed across developmental stages and across cell types, especially since some genes, including ion channels, are expressed in multiple cell populations.

It is also important to recognize that the development of functional circuits lags behind the establishment of anatomical connectivity, resulting in transient stages of neuronal activity that are either absent or may be considered pathological in adulthood. The field of developmental systems neurosciences, which integrates the genetic, molecular, and cellular perspectives of developmental neurosciences with the functional perspective of systems neuroscience, will be essential to advancing our understanding.

In the early stages of brain development, particularly during embryonic stages, cell migration occurs while others begin to differentiate. Crosstalk between different cell populations regulates this process through both cell–cell contact and through the release of signaling molecules. For example, bidirectional communication between migrating cortical interneurons and the intermediate progenitors of projection neurons controls the recruitment of the former and the proliferation of the latter, thus influencing the final number of projection neurons in the upper layers of the cortex [258]. Early generated interneurons (EGins) produced before E10.5, are predominantly SOM+ and exhibit exceptionally extensive axonal arborization. In the CA3 region, these EGins become functional hub neurons capable of influencing giant depolarizing potentials and, therefore, contributing to entorhinal–hippocampal circuit maturation [259]. In the neocortex, stimulation of an EGin influences spontaneous network synchronization, and their ablation reduces spontaneous synchronization, as well as the formation of inhibitory synapses during the first postnatal week [260]. Furthermore, the optogenetic enhancement of upper layer pyramidal neuron activity in the prefrontal cortex at neonatal stages promotes premature dendritic growth, affects interneurons density, and alters the excitation/inhibition balance in the prefrontal circuit [261]. A growing body of evidence underscores the critical role of glial and neuroglial interactions in cortical development and network function. For example, GABA-receptive microglia interact with inhibitory synapses during development and shape inhibitory connectivity without affecting excitatory synapses [262]. Calcium activity in astrocytes precedes the spontaneous switch of cortical circuits to a slow oscillation state characterized by synchronized neuronal firing, and the optogenetic activation of astrocytes releases extracellular glutamate which is necessary for this switch, highlighting their involvement in the regulation of cortical synchrony [263]. Additionally, myelination by oligodendrocytes represents another powerful mechanism for regulating circuit function with significant variation in myelination time course across different brain regions [264]. Stimulating circuit activity enhances de novo myelination via the production of new oligodendrocytes while the remodeling of existing myelin sheaths can alter axonal conduction properties without the requirement of newly produced oligodendrocytes or myelin. Indeed, just altering the length of existing myelin sheaths could modify myelin coverage along the axon and the distance between nodes of Ranvier [265].

It is essential to remember that there are more than subtle differences between rodent and human brains. This is particularly evident in the increased susceptibility of homo sapiens to neurological disorders. Evo-devo studies have revealed distinct features in humans, such as the expansion of cortical, cerebellar, and white matter structures, as well as enhanced dendritic arborization and increased spine density [266]. Human astrocytes are also much larger, more complex, and more heterogeneous than those in mice [267]. Comparative analysis of different primates’ brains by single cell sequencing has identified numerous species-specific variations in many regions. In the prefrontal cortex, these variations include cell-type specific expression shifts (hundreds of layer-markers switched their expression to another layer, not limited to neuronal markers), species-specific cell subtypes (five cell subtypes exist only in a subset of primate species), and human-specific gene expression (for example *CACNA1D* in microglia) [268,269]. In the middle temporal gyrus, human astrocytes show more differentially expressed genes with more divergent expression and are notably enriched in genes involved in synaptic signaling and translation pathways. As an example, three glutamate AMPA receptor subunits are expressed at more than threefold higher levels in human astrocytes, indicating an enhanced responsiveness of these cells to glutamate [270]. Finally, human oligodendrocytes have undergone accelerated gene expression evolution compared with neurons and their human-specific networks are enriched for alternative splicing and transcriptional regulation but also for variants associated with schizophrenia and other neuropsychiatric disorders [271].

While humans have specific characteristics that cannot be reproduced in animal models, brain tissue from IGE patients cannot be obtained due to obvious ethical constraints. In this context, brain organoids, i.e., self-organizing three-dimensional tissues derived from induced pluripotent stem cells (e.g., iPSCs) represent a major breakthrough. Human thalamic organoids (hThOs) have been successfully generated and fused with cortical organoids (hCOs) to form “assembloids” (hThCOs) that mimic reciprocal projections, i.e., TC and CT projections. These projections do not invade each organoid randomly but tend to target more specific regions. Moreover, synaptogenesis occurred in both types of projections and the thalamic neurons display distinct electrophysiological properties when recorded in hThOs or in hThCOs [272]. This model allows for the opportunity to study the development of the human thalamus and the reciprocal connectivity between thalamus and cortex. As such, these assembloids have been used to investigate the consequences of either gain- (GOF) or loss- (LOF) of-function mutations in *CACNA1G* [273]. Although these mutations do not influence the patterning or cell composition of hThOs, they do increase spontaneous firing rates of neurons in both the heterozygous or homozygous forms. The CACNA1G GOF mutation induced correlated hyperactivity in hThCOs, whereas the LOF mutation increases the number of projections without causing hyperactivity [273]. Very recently, ventral thalamic organoids (vThOs) with thalamic patterning including the nRT were obtained [274]. Neurons in vThOs exhibit burst firing like those observed in nRT from animal models [274]. Surprisingly, the deletion of *PTCHD1* or *ERBB4* does not affect lineage specification but does alter neuronal firing pattern by prolonging the inter-spike interval [274].

Despite these promising advances, organoids still resemble a developing brain rather than a mature adult brain. We are, therefore, far from studying the mature function of neurons with this approach, and even if they can generate spontaneous network activity, the predictive validity of assembloids is difficult to assess because there is no evidence of spontaneous seizures in these structures. However, twenty years ago it would have been unimaginable to study human neuron development and differentiation or the effects of gene mutations at this level of detail. There is still a long way to go with many challenges ahead, but the hope of developing new human-relevant models that reproduce more complex phenotypes and encompass mature neuronal networks now seems within reach.

Nevertheless, animal models of IGE will continue to provide valuable insights into the underlying pathophysiological mechanisms of these epilepsies, contributing to the development of more effective treatments for human syndromes.

These models are particularly useful for testing novel AED that are designed to target specific contributors to the epileptic process with greater efficiency and specificity, thereby potentially minimizing the major side effects associated with current AED. For example, Z944—a novel, highly selective pan-antagonist of inactive T-type calcium channels—has been validated in GAERS and has now reached phase II clinical trials for the treatment of absence epilepsy [275]. This compound demonstrates superior potency (active at nanomolar concentrations) and specificity in blocking T-type calcium channels when compared with ETX, which may also act on other proteins such as GIRK channels, L-type calcium channels, non-inactivating Na+ currents, IRK1, and Ca^2+^-dependent K+ currents [276,277].

Furthermore, studies suggest that genetic factors may underlie the variability in patients’ responses to AED [278,279]. This highlights the need to identify specific genes and other contributing factors, such as epistasis and gene–environment interactions. In this context, animal models of IGE represent a valuable tool for unraveling these complex relationships.

Similarly, animal models provide unique opportunities to study epileptogenesis and to test AED or other types of treatment at various stages of the process with the aim of preventing seizure development. If successful, such an approach could be applied to personalized medicine through human genomics once our understanding of the genetic architecture of epilepsies is sufficiently advanced to predict specific epilepsy syndromes, an achievement that remains out of reach for IGE currently.

Lastly, a significant portion of IGE patients are drug resistant which has a considerable impact on their everyday quality of life. Neurostimulation approaches, such as deep brain stimulation (DBS) and responsive neurostimulation (RNS) targeting the ANT or the centromedian thalamic nucleus (CMT), are emerging as promising treatments for these refractory cases [280]. Chronic stimulation of the CMT has been shown to reduce the frequency of generalized seizures (AS or GTCS) with several patients becoming seizure-free and some experiencing more than a 50% reduction in seizure frequency [281,282]. Animal models can be used to test different brain regions and stimulation protocols to try to improve their efficiency. Optogenetics is one such emerging technology that offers unprecedented specificity, enabling the precise manipulation of neuronal activity (activation or inhibition) in time, space, and cell type. Although significant progress has been made, translational challenges remain. These include the practical limitations of light delivery and the targeting of light-sensitive channels to specific brain regions. In the meantime, preclinical models will be invaluable for assessing the therapeutic potential of the modulation of specific brain nuclei and even specific cell types within these areas using optogenetic approaches.

## Figures and Tables

**Table 1:** Animal models of idiopathic generalized epilepsies described in the manuscript and their epileptic phenotype.

Species	Strain	Gene	Mutation	Seizure Description
**Absence epilepsy**				
*Spontaneous models*				
Mouse	tg	Cacna1a	P601L	Spontaneous SWDs. Ataxia
Mouse	stg	Cacng2	Transposon	Spontaneous SWDs. Ataxia
Mouse	lh	Cacnb4	4nt insertion	Spontaneous SWDs. Severe ataxia
Mouse	swe	Nhe1	K442X	Spontaneous SWDs. Ataxia
Rat	GAERS	Cacn1h/Polygenic	R1548P	Spontaneous SWDs
Rat	WAG/Rij	Unknown/Polygenic	-	Spontaneous SWDs
*Genetic manipulation of candidate genes*				
Mouse		Gabra3	Deletion	No spontaneous SWDs
Mouse		Gabrb3	Deletion	Rare spontaneous SWDs
Mouse		Gabbr1	Deletion	Rare spontaneous SWDs
Mouse		Gabbr2	Deletion	No spontaneous SWDs
Mouse		Gat-1	Deletion	Spontaneous SWDs
Mouse		Cacna1g	Deletion	No spontaneous SWDs. Resistant to GHB, Baclofen
Mouse		Cacna1g	Overexpression	Spontaneous SWDs
Mouse		Cacna1i	Deletion	No spontaneous SWDs. Increased sensitivity to GHB
Mouse		Cacna1h/1i	Deletion	No spontaneous SWDs. Increased sensitivity to GBL
Mouse		Hcn1	Deletion	No spontaneous SWDs
Rat		Hcn1	Deletion	Spontaneous SWDs
Mouse		Hcn2	Deletion	Spontaneous SWDs
Mouse		Hcn4	Deletion	No spontaneous SWDs
**Juvenile Myoclonic Epilepsy**				
*Spontaneous models*				
Baboon	Hamadryas	RBFOX1	SNP	Spontaneous myoclonus and GTCS
Dog	Ridgeback	DIRAS1	4nt deletion	Myoclonic jerks and GTCS
*Genetic manipulation of Mendelian genes*				
Mouse		Gabra1	Deletion	Myoclonic seizures, PSD, absence seizure
Mouse		Gabra1	A322D	Myoclonic seizures, PSD
Zebrafish		gabra1	Deletion	Light induced seizure inhibited by VPA
Mouse		Efhc1	Deletion	Spontaneous myoclonus. Increased sensitivity to PTZ
Fly		Defhc1.1	Deletion	Not studied
Mouse		Gabrd	Deletion	Increased sensitivity to PTZ
Mouse		CaSR	Deletion	Not studied
Mouse		Brd2	Deletion	Spontaneous seizures in some females and increased sensitivity to flurothyl in both sexes
Mouse		Cilk1/Ick	Deletion	Tonic–clonic and PSD under isoflurane
*Genetic manipulation of other mutated genes*				
Zebrafish		Cpa6	knockdown	Resistance to PTZ and pilocarpine induced seizures
Fly		Oatp308	Knockdown	Hyperthermia seizure-like and vibration over-reaction
**Generalized Tonic–Clonic Seizures Alone epilepsy**				
*Spontaneous models*				
Rat	NER	Phf24	Retrovirus insertion	GTCS

SWDs: spike and wave discharges; PSD: polyspike discharges; GTCS: generalized tonic–clonic seizure; GHB: γ-hydroxybutyrate; GBL: γ-butyrolactone; VPA: valproic acid; PTZ: pentylenetetrazol; SNP: single nucleotide polymorphism; nt: nucleotide.
